# Extracorporeal Membrane Oxygenation Use in Fat Embolism Syndrome: Current Perspectives and Clinical Insights (A 20-Year Review)

**DOI:** 10.3390/jcm14041318

**Published:** 2025-02-17

**Authors:** Ali Al Bshabshe, Wesam F. Mousa, Nashwa Nor El-Dein, Mohamed W. Mousa

**Affiliations:** 1 Department of Medicine/Adult Critical Care, College of Medicine, King Khalid University, Abha 61413, Saudi Arabia; 2 Department of Anesthesia/Adult Critical Care, College of Medicine, Tanta University, Tanta 31512, Egypt; wesammousa@hotmail.com; 3 Department of Medicine, College of Medicine, Tanta University, Tanta 31512, Egypt; nashwanor@hotmail.com (N.N.E.-D.); mohammedwesammousa@hotmail.com (M.W.M.)

**Keywords:** ECMO, extracorporeal life support, fat embolism syndrome, FES

## Abstract

**Background/Objectives**: Fat embolism syndrome (FES) is a rare but serious complication that may arise after long bone fractures, characterized by fat globules entering the bloodstream and causing multi-organ dysfunction, particularly respiratory failure. While initial treatment focuses on supportive care including oxygen therapy, fluid management, and hemodynamic stabilization, severe cases may require advanced life support, such as extracorporeal membrane oxygenation (ECMO). The aim of this study is to evaluate the role of ECMO in managing severe cases of FES with refractory hypoxemia and hemodynamic instability by analyzing patient outcomes. **Methods**: This mini-review explores the role of ECMO in managing FES by analyzing published case reports from the first documented case in 2004 and reviewing the broader literature. By examining the physiological principles, clinical applications, and reported outcomes of ECMO in FES, we aim to provide insights into its potential benefits and limitations. **Results**: A total of 12 case reports were identified and evaluated for eligibility. All 12 cases met the inclusion criteria and were included in the analysis. These cases involved patients who had FES-related refractory hypoxemia and required ECMO support. **Conclusions**: Our analysis of case reports presents supportive evidence that incorporating ECMO into the management of FES serves as a crucial bridge to recovery for patients who do not respond to conventional therapies.

## 1. Introduction

Fat embolism (FE) and FES are two related but distinct clinical entities that are frequently confused due to overlapping terminology. They are commonly seen after long bone fractures, particularly of the femur and pelvis, but less frequently after liposuction, severe burns, pancreatitis, and bone marrow procedures. FE refers to the mere presence of fat globules in the vasculature, which is usually asymptomatic and discovered incidentally with little clinical impact. FES, on the other hand, represents the pathological escalation of FE into a severe, potentially fatal condition caused by a systemic inflammatory response triggered by fat emboli [1,2]. Clinically evident FES occurs in approximately 1.7 of individuals with detectable fat embolism [3].

The pathophysiology of FES is highly complex, involving a combination of mechanical obstruction by fat droplets and a biochemical inflammatory response. Mechanically, fat globules occlude the microvasculature, while biochemically, the hydrolysis of fat into free fatty acids initiates a systemic inflammatory cascade. These free fatty acids inflict significant endothelial damage and provoke the release of pro-inflammatory cytokines, including TNF-α, IL-1, and IL-6, resulting in increased vascular permeability, tissue edema, and multi-organ dysfunction. This gradual inflammatory process, coupled with the conversion of neutral fats into toxic free fatty acids, explains the delayed onset of symptoms, which typically manifest 24–72 h after the precipitating event. In addition, fat emboli activate the complement system, amplifying systemic inflammation and contributing to coagulation abnormalities. Fat droplets further promote platelet aggregation and thrombus formation, compounding vascular occlusion and tissue hypoxia. These interlinked mechanisms contribute to severe complications, such as acute respiratory distress syndrome (ARDS) and multi-organ dysfunction [2].

The diagnosis of FES relies on a combination of clinical, laboratory, and radiological findings, as no single test is definitive [4]. Clinically, FES is often suspected based on the classic triad of respiratory distress, neurological dysfunction, and a petechial rash, although not all components may be present [5]. The diagnosis of FES can be supported by Gurd’s and Schonfeld’s criteria. Gurd’s criteria require at least one major (respiratory insufficiency, neurological impairment, or petechial rash) and four minor criteria (e.g., fever, tachycardia, anemia, thrombocytopenia, or lipiduria). Schoenfeld’s criteria assign points to clinical findings, with a score of 5 or more being diagnostic. Key features include petechial rash (5 points), hypoxemia (3 points), pulmonary infiltrates (4 points), fever, tachycardia, and confusion (1 point each). Both systems are valuable but often supplemented with imaging and laboratory findings for confirmation. Laboratory findings, such as hypoxemia, characterized by an arterial partial pressure of oxygen (PaO_2_) < 60 mmHg, anemia, thrombocytopenia, and elevated erythrocyte sedimentation rate (ESR), support the diagnosis [2,4]. Radiologically, chest X-rays and pulmonary CT imaging may reveal diffuse interstitial infiltrates or ground-glass opacities [6], while cerebral MRI can show characteristic findings such as the “starfield pattern” in cases of cerebral fat embolism [7]. Diagnostic challenges of FES are particularly pronounced in non-orthopedic trauma cases due to the non-specific nature of clinical manifestations and the absence of standardized diagnostic protocols, making early detection reliant on a high level of clinical suspicion [8].

Treatment for FES is predominantly supportive, focusing on oxygen therapy, ventilatory assistance, hemodynamic stabilization, and, if appropriate, early fracture fixation [1,2]. However, in severe cases of refractory hypoxemia, extracorporeal membrane oxygenation (ECMO) has emerged as a potentially life-saving intervention that has the potential for improving survival rates [9]. ECMO provides advanced respiratory and circulatory support, helping to alleviate hypoxia, stabilize hemodynamics, and create a critical window for recovery and definitive interventions such as fracture fixation [10,11]. The two key ECMO modalities are veno-venous ECMO (V-V ECMO), which focuses on oxygenation, and veno-arterial ECMO (V-A ECMO), which provides combined oxygenation and circulatory support. Indications for ECMO include severe acute respiratory distress syndrome (ARDS), cardiogenic shock, cardiac arrest, and hypoxemia unresponsive to conventional ventilation strategies [12], while contraindications encompass irreversible multi-organ failure, advanced malignancy, and uncontrollable bleeding [13]. Despite its life-saving potential, ECMO carries significant risks, including systemic inflammatory responses, hemorrhage, and renal dysfunction, requiring a careful evaluation of its risks versus benefits [14].

This mini-review explores the role of ECMO in managing FES by analyzing published case reports from 2004 to the present, highlighting its physiological principles, clinical applications, and reported outcomes.

## 2. Materials and Methods

This mini-review summarizes data from all case reports that address the use of ECMO to treat refractory hypoxemia in FES. A comprehensive search of the literature was performed across multiple medical databases, including PubMed, Scopus, and Embase, using keywords such as “extracorporeal membrane oxygenation”, “pulmonary fat embolism”, “fat embolism syndrome”, “ARDS”, and “refractory hypoxemia”.

We conducted a comprehensive review of case reports published between January 2004 and December 2024 that detailed the use of ECMO in patients diagnosed with FES. Inclusion criteria encompassed studies with explicit FES diagnosis based on established clinical or imaging criteria, detailed ECMO application, and clear patient outcomes. We excluded reports lacking sufficient procedural or outcome details and non-English publications.

Data extraction was performed independently by two reviewers, focusing on patient demographics (age, gender), trauma type, ECMO mode (veno-venous or veno-arterial), ECMO duration, survival outcomes, and complications. The primary outcome was survival after ECMO therapy, with secondary outcomes including time to ECMO initiation, complications during ECMO, and recovery status after ECMO discontinuation. Where possible, changes in oxygenation indices (PaO_2_/FiO_2_ ratio) before and after ECMO were summarized. A pooled analysis of the ECMO duration, complications, and outcomes across case reports was not conducted due to the variability in case details; however, descriptive analysis was used to identify trends and common findings in the use of ECMO for FES.

All data were entered using Microsoft Excel and analyzed using Stata version 17. Patient characteristics were summarized using descriptive statistics, with the mean, standard deviation, and range reported for continuous variables, and proportions for categorical variables.

## 3. Results

**Figure 1:**
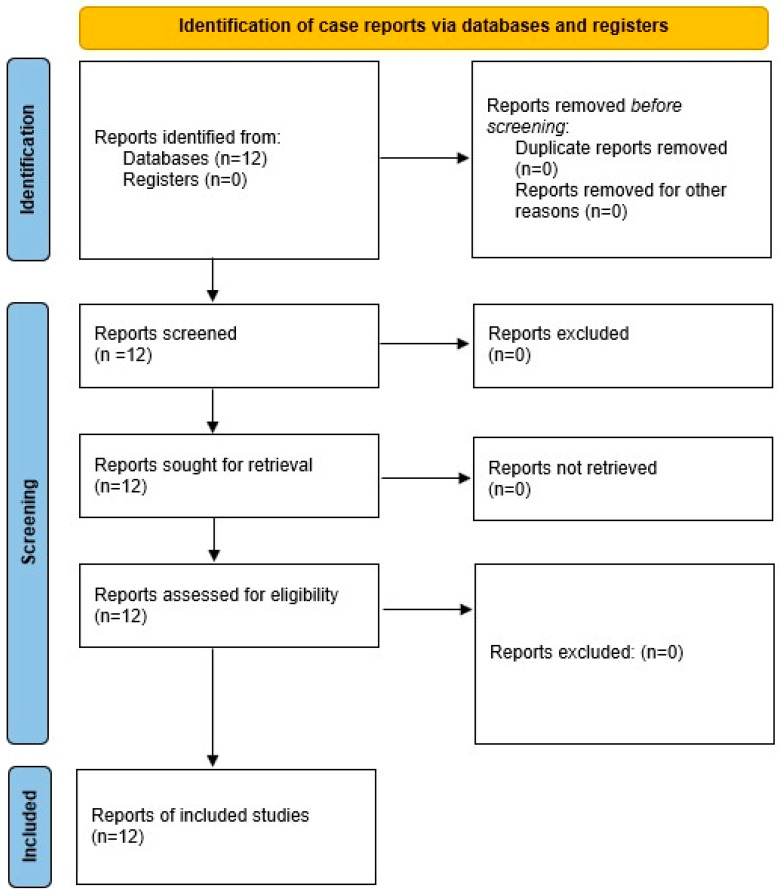
Flow chart of selection process.

Twelve cases of FES treated with ECMO over a 20-year period were identified through database searches (Table 1), and after screening, all met the inclusion criteria for review and were included in the final analysis (Figure 1). As outlined in Table 2, the cohort had a mean age of 51.08 ± 24.49 years (range: 24–90), with 8/12 (66.66%) of the patients being male. Only two cases explicitly reported proning, but all patients received lung-protective ventilation before ECMO. Both V-A and V-V ECMO were utilized equally, with each mode used in six (50%) cases. The mean time from the onset of refractory hypoxemia to ECMO initiation was 13.8 ± 5.3 h, and the mean ECMO duration was 136.08 ± 73.31 h (range: 65–288). ECMO therapy led to a significant improvement in oxygenation, with the mean PaO_2_/FiO_2_ ratio increasing from 69.7 ± 15.4 to 205.5 ± 19.2 post-ECMO, representing a +135.8 improvement. Complications were reported in 9 out of 12 (75%) cases, while survival following ECMO therapy—the primary outcome—was achieved in 11 cases; the remaining patient did not survive due to complications, including disseminated intravascular coagulation and ECMO circuit failure.

ECMO-related complications are outlined in Table 3. The most common complications were acute kidney injury (25%), ventilator-associated pneumonia (16.7%), and hemolysis (16.7%). Acute right ventricular failure and cardiogenic shock were also reported, particularly in patients requiring veno-arterial ECMO. Less frequent complications included oxygenator dysfunction (8.3%) and circuit thrombosis (8.3%), both of which required interventions such as oxygenator or circuit replacement. Coagulopathy occurred in 16.7% of cases, presenting primarily as bleeding complications managed with blood product support.

## 4. Discussion

This mini-review addresses a significant and underexplored topic in the field of critical care medicine, specifically the role of ECMO in managing FES. Our review of reported cases identifies ECMO as a rescue and bridge therapy for refractory hypoxemia in severe FES cases that fail to respond to conventional treatments. The duration of ECMO support ranged from 65 to 288 h, during which gradual improvements in oxygenation and hemodynamic parameters were observed. These findings align with previous studies demonstrating the efficacy of ECMO in managing severe respiratory failure, including ARDS resulting from non-FES causes [26]. For example, the CESAR trial [27] demonstrated improved survival in ARDS patients who received early ECMO intervention compared to those managed with conventional ventilation alone. Similarly, our findings emphasize the importance of early ECMO initiation to prevent the progression of multi-organ dysfunction, as supported by studies linking lower SOFA scores prior to ECMO initiation with better outcomes [28]. In our study, the duration of ECMO support varied among patients, reflecting the individualized nature of ECMO therapy. The CESAR trial [27] did not specify an optimal duration for ECMO support, as the focus was on the overall efficacy of ECMO referral. However, it is generally recognized that prolonged ECMO support may increase the risk of complications, underscoring the importance of regular assessment to determine the appropriate duration of therapy for each patient.

The results from Table 1 highlight the clinical presentations of FES cases requiring ECMO, where ARDS and severe hypoxemia were the most common indications for ECMO initiation. This underscores the necessity of recognizing FES as a potential cause of refractory respiratory failure in patients with relevant trauma or surgical history. Conventional lung-protective ventilation was administered in all cases prior to ECMO. However, proning was explicitly mentioned as being used in only two case reports [17,20]. The average time from the onset of refractory hypoxemia to the initiation of ECMO was 13.8 ± 5.3 h. This brief period highlights the necessity for increased clinical vigilance, timely diagnosis, and swift referral to ECMO centers, particularly as long bone fractures are often treated in smaller hospitals that lack advanced respiratory support. Insights were provided into the various modalities of ECMO used, with veno-venous ECMO being predominantly employed for respiratory support. This reflects current clinical practices, as VV-ECMO is the preferred modality for isolated respiratory failure, while VA-ECMO is reserved for cases involving combined respiratory and circulatory compromise. Table 3 addresses the complications associated with ECMO, which, while significant, were generally manageable with appropriate medical intervention. Acute kidney injury, ventilator-associated pneumonia, and hemolysis were the most common complications, consistent with the existing literature [29,30,31]. Mechanical complications such as oxygenator dysfunction and circuit thrombosis were less frequent but required timely resolution to avoid further clinical deterioration [32].

ECMO use in FES presents unique advantages compared to its application in massive PE or sepsis-related ARDS. In FES, favorable prognosis stems from the localized and reversible nature of lung injury caused by fat emboli with timely surgical stabilization that further enhances survival rates with an in-hospital mortality of 11.8%. Mortality varies by age: 8.3% in patients under 40, 14.6% in those aged 40–64, and 17.6% in those over 65 [33]. In contrast, ECMO in sepsis-related ARDS is associated with significantly worse outcomes, with a 30-day mortality of 33.1% that increases with severity: 10.5% in mild cases, 11.6% in moderate cases, and 18.1% in severe cases. The systemic inflammatory response, cytokine storm, and widespread organ dysfunction characteristic of sepsis contribute to its poor prognosis [34]. For massive PE, ECMO is typically used in conjunction with surgical or catheter-based thrombus removal, with outcomes heavily dependent on the success of these interventions [35]. These comparisons highlight the pivotal role of ECMO in FES, where prompt initiation provides crucial support during acute injury.

VA-ECMO is a valuable therapeutic intervention in patients experiencing severe cardiovascular collapse, including right-sided heart failure and refractory cardiogenic shock secondary to FES. In FES, microvascular obstruction caused by embolized fat particles leads to pulmonary hypertension, increased right ventricular (RV) afterload, and subsequent RV failure. This progression can culminate in hemodynamic instability and multi-organ dysfunction. VA-ECMO provides circulatory support by bypassing the failing heart, reducing RV workload, and ensuring adequate systemic perfusion. Similarly, in refractory cardiogenic shock following FES, myocardial dysfunction may arise from severe hypoxia, systemic inflammatory response, and direct toxic effects of embolized fat, leading to inadequate cardiac output and end-organ hypoperfusion. VA-ECMO mitigates these effects by maintaining hemodynamic stability, improving oxygen delivery, and allowing myocardial recovery [36,37].

The literature highlights not only the clinical outcomes of ECMO but also the significant logistical and practical challenges associated with its implementation. Key hurdles include the requirement for specialized personnel, advanced equipment, and high costs, which are particularly burdensome in resource-limited settings [38,39,40]. Effective anticoagulation management is especially critical in trauma-associated cases, as the bleeding tendencies and coagulopathy inherent to FES complicate protocols, demanding a careful balance to mitigate both bleeding and thrombotic risks [41]. Fat embolism can lead to complications affecting ECMO membrane functionality, particularly through the formation of lipid deposits within the oxygenator. This can result in an elevated transmembrane pressure gradient, which may require urgent membrane exchange. Such lipid accumulation could be caused by the dislodging of fat during the cannulation process, highlighting a potential risk to ECMO membrane lifespan [42]. Given these potential complications, the regular inspection of the ECMO membrane is crucial in patients with FES. Lipid accumulation within the oxygenator may not only impair gas exchange but also contribute to thrombotic deposition, further compromising membrane function. Notably, membrane clotting can occur even in patients with adequate anticoagulation, possibly due to interactions between lipids, fibrin, and platelets. This underscores the need for the close monitoring of transmembrane pressure trends and the early recognition of membrane dysfunction, as timely intervention whether through circuit modification or membrane exchange could be critical in preventing ECMO failure [43].

Patient selection further adds complexity, as individuals with advanced age, significant comorbidities, or contraindications may not be suitable candidates for ECMO [44]. Additionally, the nonspecific and variable presentation of FES often delays diagnosis and ECMO initiation, which can compromise its efficacy [45]. Existing diagnostic tools, such as Gurd’s and Schoenfeld’s criteria, provide some guidance but are limited by their reliance on clinical features that may be absent or nonspecific. Further research to develop clinical scoring systems that integrate laboratory results, imaging findings, and diagnostic biomarkers could significantly improve diagnostic accuracy and patient outcomes.

The relatively small sample size of this study limits the generalizability of its findings. Furthermore, the literature tends to exhibit a publication bias favoring positive outcomes, as cases involving ECMO use in FES with unfavorable or fatal results may be underreported. This potential bias could influence the overall interpretation of the data. In addition, this study is based on published case reports, which inherently vary in the depth of reporting, applied diagnostic criteria, methodologies, timing and method of intervention, case severity, and associated comorbidities. The retrospective nature of the review further restricts the inclusion of broader datasets or prospective comparisons that could strengthen the analysis but are beyond the scope of this work. These limitations emphasize the need for standardized reporting and larger case series to better elucidate ECMO’s role in managing this rare but severe condition.

## 5. Conclusions

Our case analysis presents possible supportive evidence that incorporating early ECMO into FES management may serve as a bridge for recovery for patients who do not respond to conventional therapies. Further studies should be performed to clarify the ECMO result in this selected population.

## Figures and Tables

**Table 1:** Fat embolism syndrome (FES) cases treated with ECMO (20-year review).

No	Study	ECMO Mode	Indication of ECMO	Age (Years)	Gender	Underlying Condition(s)	Trauma Type	ECMO Duration (Hours)	Survival Outcome	Post-ECMO Recovery	Complications
1	Egashira, T. et al. [15].	VA	Right ventricular failure	90	Female	Hypertension, dementia	Femoral fracture	168	Survived	Discharged after 13 days; no significant neurological deficits	Mild acute heart failure, ARDS
2	Ota, T. et al. [16].	VA	Right ventricular failure	86	Male	Hypertension, type 2 diabetes, pacemaker implantation	Fall, femoral trochanteric fracture	72	Survived	Recovered consciousness; extubated by Day 5; discharged to rehab by Day 59	None reported
3	Haider, S. et al. [17].	V-V	Refractory hypoxemia	25	Female	Seizure disorder	Trauma (MVA with multiple fractures)	96	Died	N/A	DIC, acute kidney injury, ECMO circuit failure
4	Yamafuji et al. [18].	VA	Refractory cardiogenic shock	79	Female	None reported	Multiple trauma (traffic accident)	72	Survived	Transferred to rehab on Day 29	None directly related to ECMO
5	Guo, P. et al. [19].	VA	Right ventricular failure	59	Male	None reported	Trauma (bike accident)	65	Survived	Discharged after 18 days; stable cardiopulmonary function	ARDS, shock
6	Momii, K. et al. [20].	V-V	Refractory hypoxemia	24	Male	None reported	Bilateral femoral and tibial fractures (traffic accident)	264	Survived	Full recovery; ventilator removed after 28 days	Hemolysis, severe acute kidney injury
7	Ballesteros, M.A. et al. [21].	V-V	Refractory hypoxemia	65	Female	None reported	Hit by a car; fractures (ribs, transverse processes, sacrum, pubis, acetabular rim), pulmonary contusion, liver laceration, and coagulopathy due to massive hemorrhage	80	Survived	Improved oxygenation; weaned from ECMO on Day 4; tracheostomy required; transferred to ward	None directly related to ECMO; coagulopathy managed with temporary anticoagulation reversal
8	Schwalbach et al. [22].	VA	Refractory cardiogenic shock	39	Male	None reported	Sub-trochanteric fracture with fat embolism syndrome	144	Survived	Discharged home with minor physical activity limitations; full RV recovery on follow-up	Acute right ventricular failure, cardiogenic shock, pulmonary hypertension
9	Popovich, I. et al. [10].	V-V	Refractory hypoxemia	24	Male	None reported	Long bone fractures (MVA)	288	Survived	Discharged neurologically intact with no residual medical issues after 36 days	Ventilator-associated pneumonia, fat deposition in oxygenator requiring circuit change
10	Wu et al. [23].	VA	Refractory cardiogenic shock	52	Male	None reported	Massive pulmonary embolism post-surgery for tibiofibular fractures	120	Survived	Discharged on day 42 with recovered renal function and intact neurological status	Renal failure, HIT, infections (surgical site and pulmonary)
11	Valchanov, K. et al. [24].	V-V	Refractory hypoxemia	32	Male	None reported	Traumatic bilateral below-knee amputations	144	Survived	Successful respiratory recovery; discharged home	None reported
12	Webb, D.P. et al. [25].	V-V	Refractory hypoxemia	38	Male	None reported	Trauma (struck by I-beam); fractures (right ulna and femur)	120	Survived	Uneventful recovery; discharged after 10 days	None reported

**Table 2:** Summary of patient outcomes and ECMO characteristics.

Parameter	Value
Number of cases analyzed	12
Mean age (years)	51.08 ± 24.49 (range: 24–90)
Gender	Male: 8 (66.66%); Female: 4 (33.33%)
Number of cases with lung protective ventilation prior to ECMO	12
Number of cases with proning prior to ECMO	2
ECMO type	Veno-arterial: 6 (50%); Veno-venous: 6 (50%)
Mean time to ECMO initiation	13.8 ± 5.3 h
Mean ECMO duration	136.08 ± 73.31 h (range: 65–288)
Mean PaO_2_/FiO_2_ before ECMO	69.7 ± 15.4
Mean PaO_2_/FiO_2_ after ECMO	205.5 ± 19.2
Improvement in PaO_2_/FiO_2_ ratio	+135.8
Number of cases with reported complications	75% (9/12)
Primary outcome (survival)	91.7% (11/12)

**Table 3:** ECMO-related complications in the study cohort.

Complication	Number of Patients (%)	Details/Occurrences
Acute Kidney Injury (AKI)	3 (25%)	Associated with prolonged ECMO duration; managed with renal replacement therapy.
Ventilator-Associated Pneumonia (VAP)	2 (16.7%)	Occurred during ECMO support; treated with targeted antibiotics.
Hemolysis	2 (16.7%)	Detected via plasma-free hemoglobin monitoring; required anticoagulation adjustments.
Oxygenator Dysfunction	1 (8.3%)	Resolved by replacing the oxygenator during ECMO support.
Circuit Thrombosis	1 (8.3%)	Managed by circuit replacement and anticoagulation optimization.
Coagulopathy	2 (16.7%)	Manifested as bleeding complications; addressed with blood products and support.

## Data Availability

Data are contained within the article.

## Abbreviations

The following abbreviations are used in this manuscript:

ARDS	Acute respiratory distress syndrome
ECMO	Extracorporeal membrane oxygenation
FES	Fat embolism syndrome
FE	Fat embolism
FiO_2_	Fraction of inspired oxygen
PE	Pulmonary embolism
PaO_2_	Arterial partial pressure of oxygen
RV	Right ventricle
V-V ECMO	Veno-venous ECMO
V-A ECMO	Veno-arterial ECMO
